# Exploring the Micro-Mosaic Landscape of *FGFR3* Mutations in the Ageing Male Germline and Their Potential Implications in Meiotic Differentiation

**DOI:** 10.3390/genes15020191

**Published:** 2024-01-30

**Authors:** Yasmin Striedner, Barbara Arbeithuber, Sofia Moura, Elisabeth Nowak, Ronja Reinhardt, Leila Muresan, Renato Salazar, Thomas Ebner, Irene Tiemann-Boege

**Affiliations:** 1 Institute of Biophysics, Johannes Kepler University, 4020 Linz, Austria; yasmin.striedner@jku.at (Y.S.); barbara.arbeithuber@jku.at (B.A.); nowak.elisabeth@outlook.com (E.N.); ronja.reinhardt@univie.ac.at (R.R.);; 2 Department of Gynecology, Obstetrics and Gynecological Endocrinology, Johannes Kepler University, 4020 Linz, Austria; thomas.ebner@kepleruniklinikum.at; 3 John P. Hussman Institute for Human Genomics, Miller School of Medicine, University of Miami, Miami, FL 33136, USA; 4 Department of Structural and Computational Biology, Max Perutz Labs, Campus Vienna Biocenter 5, 1030 Vienna, Austria; 5 Department of Physiology, Development and Neuroscience, University of Cambridge, Cambridge CB2 2EL, UK; lam94@cam.ac.uk; 6 Faculty of Science and Engineering, Anglia Ruskin University, Cambridge CB1 1PT, UK

**Keywords:** receptor tyrosine kinase, driver mutations, germline mutagenesis, congenital disorders, thanatophoric dysplasia, achondroplasia, FGFR3

## Abstract

Advanced paternal age increases the risk of transmitting de novo germline mutations, particularly missense mutations activating the receptor tyrosine kinase (RTK) signalling pathway, as exemplified by the *FGFR3* mutation, which is linked to achondroplasia (ACH). This risk is attributed to the expansion of spermatogonial stem cells carrying the mutation, forming sub-clonal clusters in the ageing testis, thereby increasing the frequency of mutant sperm and the number of affected offspring from older fathers. While prior studies proposed a correlation between sub-clonal cluster expansion in the testis and elevated mutant sperm production in older donors, limited data exist on the universality of this phenomenon. Our study addresses this gap by examining the testis-expansion patterns, as well as the increases in mutations in sperm for two *FGFR3* variants—c.1138G>A (p.G380R) and c.1948A>G (p.K650E)—which are associated with ACH or thanatophoric dysplasia (TDII), respectively. Unlike the ACH mutation, which showed sub-clonal expansion events in an aged testis and a significant increase in mutant sperm with the donor’s age, as also reported in other studies, the TDII mutation showed focal mutation pockets in the testis but exhibited reduced transmission into sperm and no significant age-related increase. The mechanism behind this divergence remains unclear, suggesting potential pleiotropic effects of aberrant RTK signalling in the male germline, possibly hindering differentiation requiring meiosis. This study provides further insights into the transmission risks of micro-mosaics associated with advanced paternal age in the male germline.

## 1. Introduction

Driver mutations promote their own propagation, as notably observed in cancer cells [1,2,3]. These mutations are well characterized in genes within the receptor tyrosine kinase (RTK)–Ras signalling pathway and its downstream branches, with an impact on cell survival or differentiation [4]. Similarly, a handful of driver mutations that affect the RTK–Ras signalling pathway have been described in the male germline. These de novo mutations (DNMs) are rare, but when they occur, they lead to sub-clonal expansion events of the mutation within the testis over time [5]. The mutant proteins have oncogenic-like properties, and a strong correlation between disease prevalence and paternal age has been documented with fathers who are significantly older than the average population [6,7], a phenomenon known as the paternal age effect (PAE) [8]. To date, the best-characterized congenital disorders associated with the PAE effect are Apert syndrome, Crouzon, Pfeiffer, the Costello and Noonan syndromes, multiple endocrine neoplasia type 2B (MEN2B), Muenke craniosynostosis, hypochondroplasia (HCH), and achondroplasia (ACH) [6,7,9,10,11]. The underlying pathogenic driver mutations are exclusive to the male germline and encode missense substitutions with gain-of-function properties, as reviewed in [12,13,14]. Given that these mutations are significant contributors to human diseases, understanding their origins and the factors influencing their occurrence, as well as their recurrence in siblings, is crucial.

In an effort to understand the molecular and biological mechanisms underlying PAE mutations and their expansion in the ageing male germline, studies assessing mutation frequencies for several PAE disorders have been conducted in sperm and testis in recent decades [15,16,17,18,19]. In sperm DNA from donors of varying ages, the frequencies of mutations causing Apert syndrome and ACH showed an increase with age [16,17,18]. Further, the examination of post-mortem testes of older donors found a localized enrichment in small regions of the testis of PAE mutations in genes such as *FGFR2, FGFR3, RET, HRAS, PTPN11, KRAS, BRAF, CBL, MAP2K1, MAP2K2, RAF1*, and *SOS1* [19,20,21,22,23,24,25,26]

This selective advantage is likely the result of changes in the RTK–Ras signalling pathway caused by a hyperactivation of the RTK signalling by the mutant protein in the male germline. Under physiological conditions, the activation of RTKs like the FGFR3 depends on growth factor (ligand) binding to the extracellular receptor domains. The conformational changes elicited by this ligand binding are transmitted inside the cell via a rearrangement of the transmembrane helices, creating an active receptor dimer (or multimer). This triggers the autophosphorylation of the intracellular kinase domains within their activation loops and subsequently the autophosphorylation of their C-terminal extensions, which are in turn recognized by downstream effector proteins (e.g., GRB2) via their phospho-peptide binding domains. This tightly orchestrated cascade of events ensures specific, high-fidelity signal transduction; hence, its corruption causes deregulation and disease [27].

It has been hypothesized that this signal hyperactivation in RTKs induces a modified division pattern in the sexually mature testis. Actively dividing spermatogonia A (SrAp) normally divide asymmetrically, resulting in an undifferentiated daughter cell and a cell that differentiates into sperm. In contrast, mutant SrAp might occasionally divide symmetrically, resulting in two daughter stem cells and a progressive clonal expansion of mutant germ cells that stay in close proximity. This can lead to a relative enrichment of mutant spermatogonia in the testis (sub-clonal expansion) or sperm equivalents, explaining the high mutation frequencies with older age [20,21,24,25,26]. A handful of mutations associated with various syndromes, such as Apert syndrome [20,21,23,24], Noonan syndrome [22,23,26], MEN2B [21], thanatophoric dysplasia I and II (TDI and TDII) [28], ACH [25], Pfeiffer syndrome [23,28], HCH, Crouzon, MEN2A, and Beare–Stevenson syndromes [23], exhibited larger clusters in testes of older donors. Conversely, young individuals (<24 years) showed minimal or no mutational clusters for Apert and Noonan syndrome, supporting the notion that clusters develop during the adult phase of male spermatogenesis, progressively growing over time [20,21].

However, there are limited data assessing both testis-expansion patterns and sperm-mutation frequencies to test the universality of the hypothesis that sub-clonal expansions in the ageing testis also result in a higher number of sperm and a higher transmission risk. Given the extensive role of RTK signalling in many developmental processes, it is likely that the biology and expansion patterns of PAE mutations might be more complex than assumed and not just the result of a selective advantage gained by SSC, but rather the interplay of several biological mechanisms affecting spermatogenesis all the way to fertilization, with some effects maybe being antagonistic. In particular, highly activating gain-of-function mutations in *FGFR3* could influence the differentiation pathway of spermiogenesis, including meiosis, and have different effects in different cell types or developmental stages.

To further examine this hypothesis, we characterized two different mutations in *FGFR3*, which are causative for congenital disorders of different severity (ACH and TDII). Both mutations uncouple FGFR3 signalling from ligand binding: c.1138G>A (p.G380R), a variant in the transmembrane helix, and c.1948A>G (K650E) that mimics phosphorylation in the activation loop of the kinase. Specifically, we examined the occurrence of these two variants in sperm samples from donors of various age groups and in an aged testis. Together with recent quantitative data comparing the signalling changes at the cell membrane of the two mutant proteins [29], we observed that the TDII mutation strongly expanded in the ageing testis, but the occurrence of the TDII mutation compared to the ACH mutation was reduced in sperm.

## 2. Materials and Methods

### 2.1. Testis, Sperm, and Control DNA

Snap-frozen post-mortem testis was obtained from the National Disease Research Interchange (NDRI, Philadelphia, PA, USA) from a 68-year-old donor free of a history of tobacco, alcohol, and drug use. The time of death after procurement was a maximum of 6 h. The donor did not have a history of cancer, chemotherapy, or radiation. Human genomic DNA (NA08859) encoding the FGFR3 c.1138G>A mutation was purchased from Coriell Cell Repositories (Camden, NJ, USA). A DNA sample heterozygous for the FGFR3 c.1948A>G mutation was kindly provided by the Greenwood Genetic Center, South Carolina. Testis and sperm samples were collected from anonymous donors and complied with the ethical regulations for the collection of human samples approved by the ethics commission of Upper Austria F-1-11.

An 1887 bp region of FGRF3 was amplified from 10 ng of human genomic DNA in a 50 µL reaction containing 0.5 µM of each primer F-ACH-88 bp, and R-TDII-BA, 1× Phusion HF Buffer (ThermoFisher Scientific, Vienna, Austria), 0.1 U Phusion Hot Start II High-Fidelity DNA Polymerase (ThermoFisher Scientific, Vienna Austria), and 0.2 mM dNTPs. The reaction was carried out with an initial heating step of 98 °C for 1 min, followed by 40 cycles at 98 °C for 15 s, 68 °C for 15 s, and 72 °C for 30 s. The 3′ A overhangs were added, with 1 U of OneTaq, to a 15 µL reaction volume that was incubated at 72 °C for 10 min. The purified amplicon was cloned into a PCR2.1 vector using the TA-Cloning Kit (Invitrogen, ThermoFisher Scientific, Vienna, Austria) and transformed into XL1-blue competent cells. The plasmid was extracted with a standard plasmid extraction protocol detailed in [30]. In short, cells were pelleted, resuspended, and lysed with an alkaline detergent solution, and the DNA was obtained through acetate/ethanol precipitation.

*Escherichia coli* (*E. coli*) DNA. XL1 *E. coli* blue cells were grown in 15 mL LB medium overnight at 37 °C until reaching an OD600 of two. A 3 mL cell suspension was then centrifuged at 8000× *g* for 30 s, and the cells were resuspended in the 10% leftover supernatant before the addition of 600 µL cell lysis solution, which is part of the Gentra Puregene Cell Kit (QIAGEN, Hilden, Germany), 24 µL 1 M DTT, and 2 µL proteinase K (QIAGEN 20 mg/mL) and incubation overnight at 37 °C. RNase treatment was performed by adding 3 µL of a 4 mg/mL RNase A solution and incubating it for 15 min at 37 °C. The reaction was put on ice for ~15 min, and 200 µL of protein precipitation solution was added by snipping the tube vigorously for 1 min, followed by two consecutive centrifugation steps at 13,000× *g* for 20 min. The DNA was pelleted by adding 600 µL of isopropanol and 1 µL of glycogen solution to the supernatant. At this step, a visible DNA bundle was formed when mixing gently. Then, the reaction was centrifuged for 30 min at 13,000× *g*, and the pellet was washed with 600 µL 70% ethanol and left to dry for 3 min. The DNA was resuspended in TE 7.5 (50 µL) overnight.

### 2.2. Testis and Sperm DNA Extraction

Testis DNA extraction was carried out as described previously [30]. In brief, for each individual testis piece using the NucleoMag Tissue Kit (Macherey-Nagel, Düren, Germany, #744300.1), according to the manufacturer’s condition except for a few modifications that ensured a mild extraction and avoided high temperatures to reduce potential sources of DNA lesions, up to approximately 20 mg of tissue was transferred into a round-bottom tube, and 100 µL of Buffer T1 and a 5 mm steel bead (QIAGEN, #69989) was added. The tissue was homogenized in the TissueLyser (QIAGEN) at 25 Hz for 1 min and then briefly spun down. The steel bead was carefully removed, and 100 µL of Buffer T1 and 25 µL Proteinase K solution (75 mg/2.6 mL) were added and mixed. The samples were incubated at 37 °C overnight. The lysed samples were then centrifuged at 6000× *g* for 5 min, and 225 µL of the cleared lysate was transferred into a low-binding DNA and RNA-free tube. Next, 24 µL of NucleoMag B-Beads and 360 µL Buffer MB2 were transferred and resuspended multiple times and incubated at room temperature for 5 min. The beads were isolated by placing the tube in the magnetic separator for 2 min, and the supernatant was discarded. A 600 µL measure of Buffer MB3 was then transferred, and the beads were resuspended in this solution, followed by an isolation step in the magnetic separator for another 2 min. The supernatant was discarded, and the beads were resuspended in 600 µL of Buffer MB4. The solution was placed in the magnetic separator, and the supernatant was discarded. Then, 900 µL of Buffer MB5 was added to the beads and incubated for 45–60 s while on the magnetic separator. The supernatant was then discarded, and the beads were resuspended with 50 µL of Buffer MB6. Following an incubation step at room temperature for 10–20 min, the beads were isolated in the magnetic separator, and the supernatant containing the genomic DNA was transferred into a low-binding nucleic acid-free tube.

Puregene Core Kit A (QIAGEN, #1042601) was used to extract genomic DNA from sperm samples according to the manufacturer’s instructions, with small alterations, as described previously in [30]. Briefly, 25 µL of each sample (∼10^6^ sperm cells) was centrifuged at 8000× *g* for 20 s, and 90% of the supernatant was discarded. The remaining 10% was resuspended, and 150 µL of cell lysis solution, 6 µL of 1 M DTT, and 0.5 µL of Proteinase K solution (20 mg/µL) were added and incubated at 37 °C overnight. Next, 0.75 µL of RNase A (4 mg/mL) was added to the previous solution and then incubated at 37 °C for an additional 15 min. Samples were placed on ice for 15 min, and 50 µL of protein precipitation solution was added and mixed thoroughly for 1 min, followed by a 4 °C centrifugation step of 20 min at 13,000× *g*. The supernatant was discarded, and the same centrifugation conditions were repeated. The supernatant was transferred to a low-binding DNA and RNA-free tube, and 150 µL of isopropanol and 0.25 µL of glycogen solution (QIAGEN, #1045724) were added. The mix was gently mixed until a DNA bundle was formed and then centrifuged at 4 °C for 30 min at 13,000× *g*. The supernatant was discarded, and the DNA pellet was washed with 150 µL of 70% ethanol, followed by a 4 °C centrifugation step of 3 min at 13,000× *g*. The ethanol was carefully removed, and the pellet was dried and resuspended in 25 µL of TE buffer (pH 7.5).

### 2.3. Bead-Emulsion Amplification (BEA)

Pre-BEA sample preparation. Testis, sperm, or plasmid DNA samples were digested with CviQI (NEB) to create ~500 bp fragments carrying the target sites. For the digest, 1200 ng of genomic DNA was digested with 6 U of CviQI (NEB), in 1× Phusion HF buffer, in a 60 µL reaction at 25 °C for 1 h, followed by overnight incubation at 16 °C.

Pre-BEA PCR. Genomic DNA targeting the loci of interest was amplified using primers F-ACH-88 bp, R-ACH-R93-SNP R2, F-TDII-3, and R-TDII-BA, with the primer sequences listed in the Supplementary Methods. Reactions were prepared to a final volume of 20 µL containing 200 ng of genomic DNA, 1× GC buffer, 200 µM of dNTPs, 1 µM of each primer, 0.5× of EvaGreen, 3% DMSO, and 0.02 U/µL of Phusion Hot Start II polymerase. The reactions were held at 94 °C for 3 min followed by 6 cycles of 94 °C for 20 s, 69 °C for 15 s, and 72 °C for 10 s and 8 additional cycles of 94 °C for 15 s, 70 °C for 15 s, and 72 °C for 10 s. Lastly, reactions were run at 72 °C for 5 min and 65 °C for 5 s as a final extension step. 

Bead preparation. To begin with, 100 µL of M-270/280 paramagnetic streptavidin-coated microbeads (ThermoScientific) were transferred into a low-binding DNA tube and placed on the magnetic particle concentrator (MPC) for 1 min. The supernatant was discarded, and the beads were resuspended in 200 µL of Bind and Wash Buffer (10 mM Tris-HCl pH 7.4, 1 mM Tris-EDTA pH 7.4, 2 M NaCl). The reactions were placed on the MPC, and the previous step was repeated 3 additional times. The isolated microbeads were resuspended and incubated in 198 µL of Bind and Wash Buffer and 2 µL of 1 mM dual-biotinylated primer (Eurofins, Munich, Germany) for 20 min (resuspended every 5 min) at room temperature, as listed in Supplementary Materials. The microbeads were then isolated on the MPC and washed twice in 200 µL of Bind and Wash Buffer, followed by two washes in 200 µL of TE Buffer (10 mM Tris-HCl pH 7.4 and 1 mM EDTA), and lastly eluted in 100 µL of TE Buffer.

BEA. This procedure follows the exact protocols described previously in [31] and initially described in [32,33] and elaborated in the Supplementary Methods. Specifically, 1 µL of a 1:10 dilution of the pre-BEA PCR reaction was hybridized to 6 × 10^6^ beads covered with the Dual-biotinylated primer (see the previous section) in 1× TitatinumTaq Buffer and 16 mM MgCl_2_. The hybridization was carried out at 94 °C for 2 min, then 80 °C for 5 min, followed by 15 min of exposure until it cooled down to room temperature (0.1 °C increments per second). The beads were washed once with water and mixed with the aqueous PCR phase with the same primers as in “Pre-BEA PCR” section and the oil phase, as described in the Supplementary Methods and in [31] and initially described in [32,33].

The emulsion was prepared using the Dow-Corning components as described in Supplementary Methods and in [31] and pipetted out in 8-PCR strip tubes. The emulsion PCR was carried out in a standard thermocycler with an initial denaturation step of 94 °C for 2 min followed by 55 cycles at 94 °C for 15 s, 65 °C for 15 s, and 72 °C for 35 s and with an end step at 72 °C for 2 min. Beads were washed and labelled as described previously in [31] with the probes targeting the site of interest with an initial denaturation step of 95 °C for 2 min followed by 60 °C for 5 min and 72 °C for 5 min and kept at 75 °C until unextended probes were washed off. Samples were then immobilized on a microscope slide and scanned as described in the Supplementary Methods and in [31] and initially described in [32,33]. In each experiment, only ~10% of the beads amplified a product given the low input ratio of DNA molecules to beads to ensure that most of the beads were attached to only one initial molecule based on the Poisson distribution. Experiments rendering more than 30% positives were discarded and repeated considering that beads seeded with more than one molecule could result in an underestimate of the mutation number. If both a mutant and wild-type molecule were on a bead, the bead resulted in a multi-coloured bead eliminated during the analysis. 

Dye switches. After the beads were scanned with a microscope, the probes were washed off, and the beads were re-labelled with the probes of interest at 95 °C for 2 min followed by 63 °C for 5 min and 72 °C for 5 min and kept at 75 °C until unextended probes were washed off. The dye switch was performed in a hybridization chamber and in situ PCR block as described previously [25,31]. Since the beads were immobilized on an acrylamide array, it was possible to re-scan the beads without losing the positional information of each bead. The labelling probes are listed in the Supplementary Materials. 

Bead-calling. For each experiment, two sets with five images each were analyzed. Brightfield images between scans (normal scan and dye switch scan) were aligned by computing the normalized cross-correlation matrix of pairs of images in Matlab R2010a (Mathworks, Munich, Germany) as described previously [31]. In brief, the analysis of images at each raster position involved custom Matlab algorithms (https://github.com/Single-Molecule-Genetics/BEA-Matlab-Analysis, accessed on 2 January 2024). This process included correcting random shifts between images by registering them based on normalized correlation. Images requiring more than a 500-pixel alignment correction were discarded. A mask was created for bright field images using wavelet-based segmentation, and this mask was then applied to subsequent washes. Beads were identified by comparing masks, and the mean fluorescent signal intensity for each bead was extracted, along with the bead identifier and area, and saved in a text file. The text file categorized beads into four clusters (00, 10, 01, 11) based on signal intensities, aiding in the classification of beads with specific genotypes. The ‘11’ cluster represented beads fluorescing in both channels, and the ‘00’ cluster represented empty beads with no fluorescence. The classification scheme was based on a Gaussian Mixture model and normalization parameters that have been described in detail previously [32]. Wild-type beads were identified as beads classified as 1001 and mutants as 0110. 

### 2.4. Statistical Analysis

Pearson’s correlation coefficient was used to determine a linear correlation between the observed and expected VAFs for the control samples using the software OriginPro 2023 (OriginLab Corporation, Northampton, MA, USA). Spearman’s correlation coefficient was used to test the correlation between variant allele frequency (VAF) and sperm donors’ age using the software OriginPro (OriginLab Corporation). A Mann–Whitney-U test was used to compare the differences in the accumulation of mutations in sperm between ACH and TDII variants. All VAF presented in this work were Poisson corrected according to the following formula:

$$\lambda =-\ln\begin{array}{c} (1-(\frac{Mutant}{Mutant+Wild-type})\; \end{array}$$

## 3. Results

**Figure 1:**
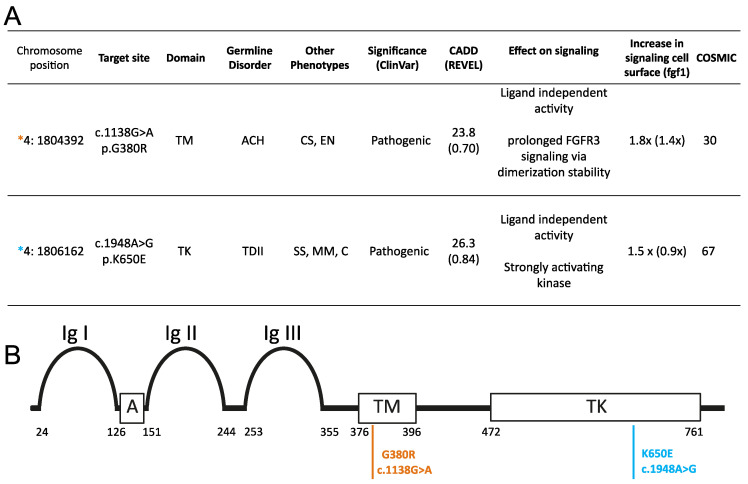
Variants and associated information. (**A**) Phenotype data, mainly retrieved from ClinVar. The effect on signalling was as reported by VarMap [50]. Predicted pathogenicity scores like the CADD (Combined Annotation Dependent Depletion) score [51] and REVEL score [52] (ranging from 0 to 1, with 1 being the most pathogenic) are according to GRCh38-v1.6 and represent the level of deleteriousness. The fold increase in the signalling of the mutant compared to the wild-type protein on the cell surface was determined without and with the addition of ligand (value in parenthesis) reported in [29]. COSMIC (Catalogue of Somatic Mutations in Cancer) data were based on version 94 [53]. All are is according to transcript ENST00000440486, *FGFR3* isoform IIIc. ACH: achondroplasia; TDII: thanatophoric dysplasia II; CS: craniosynostosis; TM: transmembrane domain. TK: tyrosine Kinase domain; fgf1: fibroblast growth factor I; SS: spermatocytic seminoma; MM: multiple myeloma; C: carcinoma; EN: epidermal nevus. The color of the asterisks matches the respective mutation in panel B. (**B**) Schematic illustration of functional domains in FGFR3. The position of the amino acid substitution associated with ACH (orange) or TDII (blue) is indicated in the respective domains.

We examined the variant allele frequency (VAF) of c.1138G>A (p.G380R) and c.1948A>G (p.K650E) in sperm donors of diverse ages and in one post-mortem dissected testis of a 68-year-old donor. The c.1138G>A (p.G380R) variant, screened in the same experiment with some data presented elsewhere [19], is associated with ACH, craniosynostosis syndrome, and epidermal nevus [34,35,36,37,38,39] and is well-characterized in the male germline. It is more prevalent in sperm from older donors [17,40], forms sub-clonal clusters in the testis [23,25], and has a higher incidence in offspring of older fathers [7,35,39]. Its deleteriousness CADD score predicted in silico is 23.8 (Figure 1), and it has been reported to exhibit moderate ligand-independent, constitutive activation of the RTK signalling pathway [19,29,41,42,43].

The pathogenic variant c.1948A>G (p.K650E) is associated with TDII and has been observed in spermatocytic seminoma and multiple myeloma cases [44,45,46]. With a CADD score of 26.3 and a substantial mutation count reported in COSMIC (Figure 1), this missense substitution is embryonic lethal already at the heterozygous state. The constitutively active TDII mutation [47,48] was reported as one of the strongest activating mutants of FGFR3 signalling in whole-cell lysates [43,49]. Yet the comparative analysis of signalling at the plasma membrane showed a similar ligand-independent activation of both mutations [29]. Both variants are classified as deleterious/pathogenic by ClinVar (Figure 1).

### 3.1. Determination of Low-Frequency Mutations in the Male Germline

**Figure 2:**
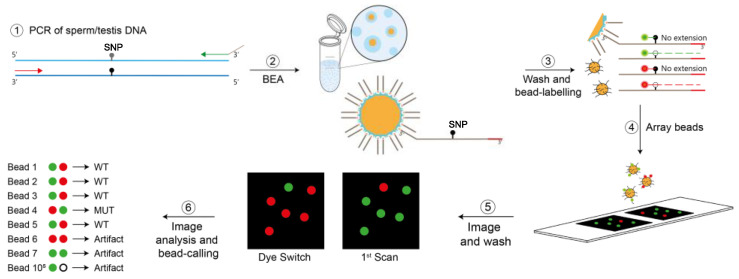
Schematics of the bead-emulsion amplification (BEA) process. In Step 1, regions including sites c.1138 and c.1948 undergo multiplex PCR from testis or sperm genomic DNA. In step 2, single amplicons are hybridized with microscopic beads covered with a dual-biotinylated primer complementary to the amplicons (overhang tail). PCR products are produced on the bead within an emulsion droplet. In step 3, the beads are washed and labelled using allele-specific extensions of fluorescent probes specific for the locus and the mutant or the wild-type (WT) site. The wild-type and mutant for c.1138 and c.1948 can be distinguished during the scanning using four different specific probes, each labelled with a different Alexa dye [33]. Only two colours are shown, as exemplified in green and red. In step 4, un-extended probes are washed off, and the fluorescent beads are arrayed on a slide. The array is scanned with an inverted fluorescence microscope (Zeiss Axio Observer.Z1, Munich, Germany) with a 20× objective followed by a subsequent washing, probing, and imaging cycle to confirm the mutants with a dye switch. A series of imaging and data analysis steps are performed to assess the mutation frequency in ~10^5^ molecules, as elaborated in [32]. Note that both sites were screened in the same experiment, but mutant and wild-type counts are measured independently for each site using the different probes. Details on the protocol steps are published in [31].

In order to determine the mutation frequencies of both *FGFR3* variants in sperm and testis DNA, we used digital PCR that can measure VAFs ≥ 10^−5^. Specifically, we used bead-emulsion amplification (BEA), a previously validated in-house digital PCR method that examines single molecules multiplexed for two sites [25,31,32,33]. A schematic of this method is shown in Figure 2.

**Figure 3:**
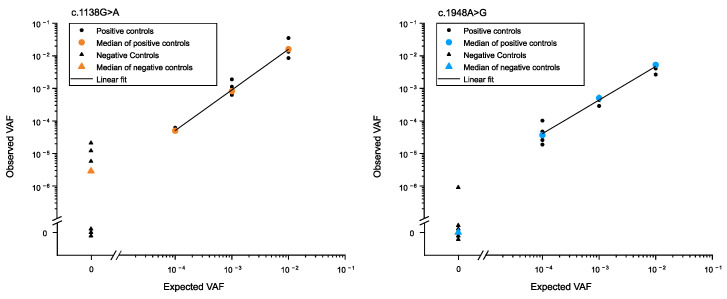
Validation of estimated ACH (c.1138G>A) and TDII (c.1948A>G) mutation frequencies measured with BEA. Different ratios of mutant to WT were reconstructed by mixing distinct amounts of genomic DNA from either an ACH or a TDII carrier DNA with WT genomic DNA. Data points represent individual and median measurements (small and larger circles, respectively). The measured ratios match the known input ratios ranging from 1 mutant to 100, 1000, and 10,000 WT with Pearson’s correlation coefficient (r), *r* = 0.99 and 0.98 for c.1138 and c.11948, respectively. Negative controls (triangles) are 300,000 copies of WT plasmid mixed with *E. coli* as carrier DNA. Note that the negative controls with an observed VAF of 0 are displayed with a slight vertical offset to prevent overlapping data points. Data for positive and negative controls can be found in Supplementary Table S1. Data on the ACH are published in [19] and are shown here only for comparative purposes.

The accuracy and reproducibility of the method were determined through a serial dilution experiment with positive controls (ACH- or TDII-carrier DNA) spiked into wild-type (WT) human genomic DNA at various ratios (Figure 3 and Supplementary Table S1). The method exhibited a good correlation between input and observed ratios of genomic DNA. A Pearson’s correlation coefficient (r) of 0.99 and 0.98 for c.1138G>A and c.1948A>G, respectively, confirmed the assay’s accuracy and reproducibility. Additionally, the background mutation level of a wild-type plasmid was measured, revealing levels below those of the positive controls (Figure 3) and reflects the method’s sensitivity to measure VAFs at 10^−5^ or larger. In this study, we utilized approximately 300,000 genomes per input, followed by PCR and emulsion amplification on beads.

### 3.2. Transmission of Mutations in Testis

**Figure 4:**
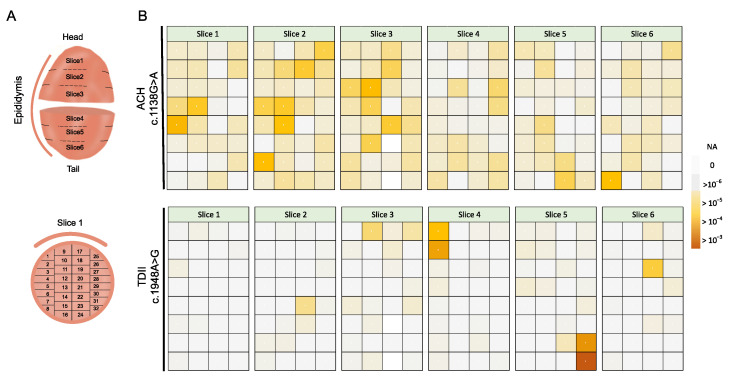
Spatial analysis of sub-clonal expansions in a human testis. (**A**) Strategy for a testis-cutting scheme as described previously [20,21,24,25]. In brief, the testis was dissected into six slices, and each slice was dissected into 32 individual pieces (total n = 192 pieces). The thin, curved line next to the testis denotes the position of the epididymis for orientation purposes. (**B**) Spatial distribution of the two variants across each testis piece. The colour-coded scheme refers to the variant allele frequency (VAF) of each piece in a range of >10^−3^ to 0. NA: Not available. The data can be found in Supplementary Table S2.

To test the sub-clonal expansion in the male gonad, we assessed the spatial distribution of the mutations in the testis of a post-mortem donor (68 years old). We adapted the testis dissection strategy used previously in combination with single-molecule PCR screening for this purpose [20,21,24,25]. Briefly, the human testis was dissected into 6 slices and further cut into 192 pieces (Figure 4A,B). Figure 4B shows the spatial distribution of c.1138G>A and c.1948A>G (data in Supplementary Table S2).

For the canonical PAE variant associated with ACH (c.1138G>A), we measured mutations in many pieces (average VAF = 3.5 × 10^−5^; median VAF = 2.3 × 10^−5^), yet some pieces accumulated more mutations than neighbouring pieces, with the highest measured VAF (MaxVAF) being 4.1 × 10^−4^, as also reported in [19]. The MaxVAF/IQR ratio, representing the highest VAF of all pieces relative to the rest of the testis, was ~12-fold larger than the interquartile range (IQR); see Table 1. In the case that mutations are uniformly distributed (no cluster formation), the MaxVAF/IQR should be closer to 1. In a different testis dissection study (also using BEA), the sub-clonal clustering was much more localized, with the MaxVAF being 65-fold higher than the IQR [25].

The TDII mutation exhibited a more extreme non-uniform distribution, forming focal mutation pockets that measured VAFs 2–3 orders of magnitude higher than adjacent pieces (Figure 4B). The MaxVAF/IQR ratio differed by over two orders of magnitude (MaxVAF/IQR ~500-fold; Table 1). The average VAF was 3 × 10^−5^, with a few individual pieces showing VAFs as high as 10^−3^. These parameters strongly support a sub-clonal expansion event for TDII, with individual pieces reaching higher VAFs than for the ACH mutation, indicating a stronger clonal expansion for TDII than for the canonical PAE mutation ACH.

### 3.3. Transmission of Mutations in Sperm 

**Figure 5:**
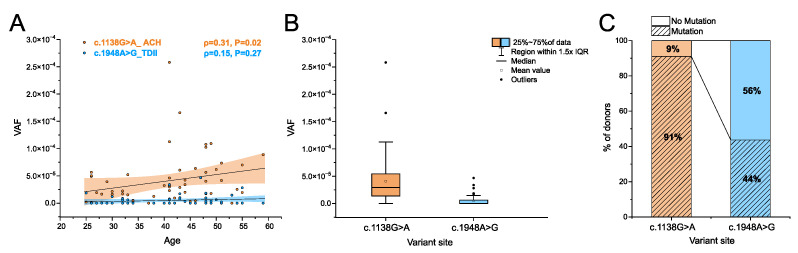
Variant allele frequencies (VAF) of the ACH and TDII mutation measured in sperm DNA. (**A**) Correlation between VAF and the donor’s age (23 to 59 years old). The coloured areas represent the confidence bands, and the black line shows the linear regression. Spearman’s correlation test (*ρ*) was used to assess a positive correlation between mutation frequency and donor’s age. The data can be found in Supplementary Table S3. Sperm data for ACH were taken from [19]. (**B**) Comparison of differences in the accumulation of mutations in sperm between ACH and TDII with the *p*-value estimated using the Mann–Whitney-U test (*p*-value < 10^−10^, Z-score = 6.8). IQR: Interquartile range. (**C**) Percentage of sperm samples without or with mutations for the two variants. The number of total donors screened for each locus was n = 56 and 55, respectively. Percentages are rounded with no decimal numbers.

We examined 55–56 sperm donors (25 to 59 years) in parallel for the ACH and the TDII mutation, respectively. As shown in Figure 5A, the ACH mutation (c.1138G>A) increased with the donor’s age, with the VAF showing a positive correlation (ρ = 0.31) measured with Spearman’s correlation test, which was significant (*p*-value = 0.02), as was also reported in [19]. The highest VAF (MaxVAF, Table 2) was 2.6 × 10^−4^, as measured in a 41-year-old sperm donor with the median VAF for all sperm donors reaching 2.9 × 10^−5^, and an interquartile range (IQR) of 5.9 × 10^−5^ (Table 2; Supplementary Table S3).

In comparison, for the TDII mutation (c.1948A>G), older sperm donors generally showed an increased mutation accumulation, as indicated by a positive correlation (ρ = 0.15), but this correlation did not reach statistical significance (*p-*value = 0.27; Figure 5A). The maximum VAF of 4.7 × 10^−5^ was measured in a 47-year-old sperm donor. The median frequency was 0, and the interquartile range (IQR) 5 × 10^−6^ was almost seven-fold lower for TDII than for the ACH mutation (Table 2). Overall, the mean, median, and IQR VAFs were an order of magnitude lower for TDII than for the ACH mutation. The difference in the mutational load between these two variants was significant when comparing all the data (Figure 5B).

We also compared the ratio of samples in which a mutation was measured to those without any mutations (Figure 5C). It should be noted that samples with no mutations might not represent a VAF of 0 but are likely within the detection limit of the Poisson distribution, for which the positive events occur approximately 60% of the time for VAF frequencies lower than 10^−5^ with the input of 300,000 genomes. Note that for the ACH mutation (c.1138G>A), most of the samples carried mutations (91%), whereas for the TDII mutation (c.1948A>G), this percentage was 44%, also suggesting that in general, the same sample had a lower VAF for TDII, often resulting in an ‘empty’ measurement or a VAF of zero.

## 4. Discussion

### 4.1. Pathogenic FGFR3 Mutations Accumulate Differently in the Ageing Male Germline

Our study, utilizing bead-emulsion amplification (BEA), contributes important insights into the accumulation of pathogenic *FGFR3* variants in the ageing male germline. The positive and negative controls showed that our measurements were accurate and sensitive for both variants, c.1138G>A and c.1948A>G, in sperm and testis DNA, although we cannot exclude the fact that data for c.1138G>A are noisier and samples with VAFs smaller than 10^−5^ might contain some false positive counts based on the behaviour of the negative plasmid controls.

Sub-clonal expansion events in the testis were observed for both the ACH (c.1138G>A) and the TDII mutation (c.1948A>G), with individual testis pieces reaching high levels, particularly c.1948A>G reaching the highest VAF at 10^−3^. Observations of sub-clonal expansion events for the c.1138G>A locus in the testis of an older donor have been reported previously using the same BEA method [25] or the sequencing of individual testis biopsies selected through the in situ screening of increased FGFR3 signalling [23]. In addition, our measurements of an average VAF of 3.5 × 10^−5^ for the c.1138G>A variant are consistent with findings in testis biopsies measuring VAFs of a similar magnitude (4.5 × 10^−5^, n = 5) from donors aged 65 to 70 years [23]. Our data also are consistent with previous reports showing an increase in the ACH mutation (c.1138G>A) in sperm DNA [17,40].

For the TDII mutation (c.1948A>G), we also observed strong clustering in the testis, which aligns with the severity of the associated clinical outcome resulting in embryonic lethality. However, contrary to the expectation that large clonal expansions in the ageing testis lead to an increase in mutant sperm in older donors, for c.1148A>G, the VAF increase with the donor’s age was modest and not significant. Interestingly, the VAFs measured in sperm for this variant were significantly lower by almost an order of magnitude than VAFs measured for the ACH mutation (c.1138G>A). The reduced birth incidence in TDII (1 in 100,000) compared to ACH (1 in 15,000–30,000) [6,38,46,54] may also be attributed to a lower number of TDII mutant sperm.

### 4.2. Activation of Tyrosine Kinase Signalling via the Mutations Associated with ACH and TDII 

It has been suggested that the expansion of different *FGFR3* variants in the male germline is driven by similar biological mechanisms, but to date, there have been limited data supporting this view. The ACH mutation c.1138G>A (p.G380R) introduces a strong negative charge in the dimerization interface of the transmembrane helices and was proposed to stabilize the active FGFR3 dimer, resulting in a ligand-independent autoactivation potential of the receptor and enhanced signalling activity [41,43,48]. In contrast, the TDII mutation c.1948A>G (p.K650E) mimics the activating phosphorylation in the kinase domain, thereby rendering the kinase constitutively active irrespective of the dimerization and growth factor signalling [47,48]. Consistently, p.K650E has been described as one of the strongest activating mutations of FGFR3 in whole-cell lysates [42,43,47,48,49].

A recent study compared different FGFR3 variants regarding their downstream signalling at the cellular surface using a combination of micropatterns and total internal reflection fluorescence (TIRF) microscopy. It measured the recruitment of the downstream adaptor protein GRB by the FGFR3 mutants c.1138G>A (pG380R) and c.1948A>G (p.K650E) with and without the addition of fibroblast growth factor (fgf) ligands fgf1 and fgf2 [19,29]. Both substitutions induced a ligand-independent recruitment at the basal state, with the ACH mutation (G380R) showing a higher level of activation than the TDII variant (K650E) [29]. Interestingly, this is in contrast to previous studies that estimated receptor activation based on the level of tyrosine phosphorylation in the respective mutant in whole-cell lysates (e.g., [43]). It is possible that the TDII mutant, which uncouples kinase activity from growth factor signalling, renders its activity also independent of the correct cellular localization and therefore does not translate into the hyperactivation of signalling at the cell surface level. Instead, the deregulated mutant FGFR3 may induce pathogenic off-target downstream signalling in the cytosol via the extracellular signal-regulated kinase (ERK) proteins inside the cell [55,56,57]. 

### 4.3. Role of RTK-MAPK in Meiotic Differentiation from Sperm into Spermatids

Spermatogenesis in the adult human testis involves a delicate balance between spermatogonial stem cell (SSC) self-renewal and differentiation, supported by interactions between the germ cell niche and various somatic cell types (Leydig, myoid, Sertoli, endothelial, macrophage), as identified through single-cell transcriptomics of a large number of testis cells [58,59]. Additionally, male germ cells undergo the intricate process of meiosis, transforming from mitotic spermatogonia to spermatocytes. This transition is characterized by significant gene expression changes during male meiosis and spermiogenesis [60].

The tyrosine phosphatase SHP2, encoded by the *PTPN11* gene, part of the downstream RTK signalling cascade, plays a key role in spermatogenesis and the maintenance of spermatogonial stem cells [61]. It is also crucial for maintaining a functional blood–testis barrier (BTB) [62]. Specifically, the absence of SHP2 (tested in SHP2-knockout mice) disrupts spermatogenesis by modifying the actin cytoskeleton, mislocalizing key junction proteins at the BTB, consequently affecting junction integrity and Sertoli cell support of spermatogenesis [62]. In addition, in SHP2 knockout mice, SSC differentiation was affected, leading to a reduction in spermatogonial number [61,63]. 

The RTK-MAPK signalling is also important in regulating the expression of numerous genes/proteins associated with meiotic recombination and synapsis. In SHP2 knockouts, the expression of meiotic proteins was suppressed and caused meiotic spermatocytes to undergo apoptosis [63]. This might explain male infertility in gain- and loss-of-function mutations in *PTPN11* associated with pathological conditions such as Noonan syndrome and LEOPARD syndrome [61,63]. SHP2 is abundant in spermatogonia but markedly decreases in meiotic spermatocytes, along with the increased expression of meiotic genes (e.g., *Dmc1*, *Rad51*, and *Smc3* were reduced in SHP2 knockouts) [58,63]. Further, it has also been shown that RTK-MAPK signalling is important in regulating the disassembly of synaptonemal complex (SC) proteins during late meiotic pachytene, with the MAPK inactivation being required for the timely disassembly of SC proteins and coordination of crossing overs [64]. Conversely, the constitutively phosphorylated SYP-2 of the SC was found to impair the disassembly of the SC and the progression of the meiotic cell cycle [64].

Together, the findings of these publications suggest that an aberrant or hyperactive RTK-MAPK signalling might have an effect on the differentiation phases from spermatogonia to spermatid formation. A study examining the different possible mutations associated with codon p.650 in FGFR3 observed that variants with significantly increased receptor kinase activity compared to the wild type [43] also had a strong expansion prevalence in somatic (e.g., skin tumors) and male germline tissue (e.g., testicular tumours, but were underrepresented in sperm [15]. However, this observation may be attributed to two plausible hypotheses: firstly, robust sub-clonal expansions may not be induced in the normal ageing testis via the strong activation of the RTK signalling, or secondly, SSC cells may not differentiate into sperm with meiosis being impaired. Note that each scenario is possible since each affects different cell types—spermatogonia or spermatocytes, respectively

Based on our study, measuring the mutation distribution in both testis and sperm, we suggest that in the context of RTK-constitutive and high signalling, only a fraction of mutant SSC differentiate into sperm. We observed that the TDII mutation (c.1948A>G) clustered strongly in the testis, aligning with its phenotypic severity, but showed only a modest and non-significant age-related increase in sperm. This contrasting mutational load in testis versus sperm is best explained by a mechanism hindering spermiogenesis interfering with meiosis in mutant spermatocytes due to aberrant RTK signalling. Also, VAFs in sperm for the TDII mutation were an order of magnitude lower than for the ACH mutation (c.1138G>A). 

A decrease in average VAFs in testis compared to sperm for activating RTK mutations can also be derived indirectly from data on the mutation associated with Apert syndrome (c.755C>G, p.S252W in *FGFR2*). This variant is located in the linker region between the IGII and IGIII domain and contributes to the FGF binding site of the receptor. Its mutation causes a loss of specificity for the activating growth factor and results in an increased unspecific activation of the receptor [65]. The average number of mutations for variant c.755C>G in *FGFR2*, measured in the testis, was 8–10 times higher than the average mutation frequency measured in sperm when comparing age groups older than 35 years (Table 3). Specifically, 18% of 168 sperm donors versus 83% of the testis donors within a middle-aged group (36–68 years) had a higher average VAF than the youngest group, eliminating the possibility of a sample size effect. This discrepancy, observed across data collected using the same method and group [18,21], also challenges the assumption that larger clusters in the testes also results in a higher mutation load in sperm.

## 5. Conclusions

In conclusion, our investigation revealed distinct behaviors for c.1138G>A and c.1948A>G variants. The observed discordance between mutation frequency in testis and sperm for these two variants underscores the intricate interplay between activation levels, ageing, and meiotic differentiation, offering a foundation for further understanding the molecular mechanisms underlying the expansion and transmission of pathogenic driver mutations in the male germline. 

## Figures and Tables

**Table 1:** Summary statistics of testis measurements of a post-mortem testis from a 68-year-old donor. Mean VAF, median, and maximum VAF (MaxVAF) are estimated from data of individual testis pieces in Supplementary Table S2. VAF: Variant Allele Frequency. IQR: Interquartile range. Max MaxVAF/IQR: Ratio of maximum mutation frequency to testis IQR mutation frequency. n: sample size. ^%^Data taken from [25].

Variant	Disorder	Age of Donor	Mean VAF	Median VAF	IQR	Max VAF	MaxVAF/IQR	n
c.1138G>A p.G380R	ACH	68	3.5^−5^	2.3^−5^	3.4^−5^	4.1^−4^	12	190
		^%^80	^%^1.3^−4^	4.0^−6^	4.7^−5^	3.1^−3^	65	^%^192
c.1948A>G p.K650E	TDII	68	3.4^−5^	0.0	6.1^−6^	3.0^−3^	485	190

**Table 2:** VAFs measured in sperm DNA donors aged 23 to 59 years old. Mean VAF, median, and maximum VAF (MaxVAF) are estimated from data of individual sperm donors listed in Supplementary Table S3. Sperm data for 1138G>A are from [19]. VAF: Variant allele frequency. IQR: Interquartile range. IQR: Interquartile mutation frequency. n: sample size.

Disorder	Variant	Mean VAF	Median VAF	IQR	Max VAF	n
ACH	c.1138G>A p.G380R	4.1^−5^	2.9^−5^	4.1^−5^	2.6^−4^	56
TDII	c.1948A>G p.K650E	4.5^−6^	0	6.0^−6^	4.7^−5^	55

**Table 3:** Mutants per million genomes (pmg) measured in different testis and sperm donors for the Apert mutation c.755C>G. % ^a^ Percentage of donors with more than 10× the mutation frequency of the youngest age group. Sperm data summarized from [18] and testis data from [21].

**Sperm Data [18]**						
**Age group**	**Average (pmg)**	**Median**	**Max**	**Min**	**Sample size**	**%** ^a^
19–23	4.5	2.1	34.2	0	23	0%
36–68	34.4	17.2	724.2	0	168	18%
75–80	16	16	31.9	0	3	33%
**Testis data [21]**						
**Age group**	**Average (pmg)**	**Median**	**Max**	**Min**	**Sample size**	**%** ^a^
19–23	5	1.5	16	1	4	0%
36–68	358.5	270	861	3	6	83%
75–80	278	166	621	25	5	80%

## Data Availability

All the data produced in this study are available in Supplementary Materials.

## Supplementary Materials

The following supporting information can be downloaded at https://www.mdpi.com/article/10.3390/genes15020191/s1. Supplementary Methods; Table S1, Control data; Table S2, testis data; Table S3, sperm data.
