# Supplementary material for: Exploring the Micro-Mosaic Landscape of FGFR3 Mutations in the Ageing Male Germline and Their Potential Implications in Meiotic Differentiation
Source: Genes (Basel). 2024 Jan 30;15(2):191. doi: 10.3390/genes15020191 (PMC10888257; doi:10.3390/genes15020191)
Supplement: Supplementary file 1 [file genes-15-00191-s001.zip › Supplementary_Methods.pdf]

Supplementary Methods

BEA: bead-emulsion amplification

Primer and probe list
Note that the last 20 base pairs of these primers (lower case) are complementary to the 3' of the dual-biotinylated primer on the magnetic beads.

Pre-BEA amplification

Name	Sequence
F-ACH-88bp	5´-GAG CTG GTG GAG GCT GAC GA-3`
R-ACH_BA	5´-aga gca gga ccc caa agg acc agc AGG CAG CTC AGA ACC TGG TA-3`
F-TDII_BA3	5´-GTG CTG GTG ACC GAG GAC AAC G-3`
R-TDII_BA	5´-aga gca gga ccc caa agg acc agc CAG GCG TCC TAC TGG CAT GA-3`

Bead preparation

Name	Sequence
Bead-ACH-TDII-SNP_R2	5´-/52-Bio//iSp9/tat gtc ttt ctc tca cat aaA GAG CAG GAC CCC AAA GGA CCA GC-3`

Aqueous phase

Name	Sequence
F-ACH-88bp	5´-GAG CTG GTG GAG GCT GAC GA-3`
R-ACH-R93-SNP_R2	5´-aga gca gga ccc caa agg acc agc CCA CCA CCA GGA TGA ACA GGA AG-3`
F-TDII-3	5´-CGG GAC GTG CAC AAC CTC GAC TAC-3`
R-TDII-BA	5´-aga gca gga ccc caa agg acc agc CAG GCG TCC TAC TGG CAT GA-3`

Labeling probes

Name	Sequence
ACH wt 488	5´-/Alex488N/AGG CAT CCT CAG C*T*A*C*G-3`
ACH mut TX592	5´-/TexRd-XN/CAG GCA TCC TCA GC*T*A*C*A-3`
TDII wt 647	5´-/Alex647N/CAC AAC CTC GAC TAC TAC*A*A*G*A-3`
TDII mut 532	5´-/Alex532N/ACA ACC TCG ACT ACT AC*A*A*G*G-3`

Dye switch labeling probes

Name	Sequence
ACH wt TX592	5´-/TexRd-XN/ AGG CAT CCT CAG C*T*A*C*G-3`
ACH mut 488	5´-/Alex488N/CAG GCA TCC TCA GC*C*A*C*A-3`
TDII wt 532	5´-/Alex532N/CAC AAC CTC GAC TAC TAC*A*A*G*A-3`
TDII mut 647	5´-/Alex647N7ACA ACC TCG ACT ACT AC*A*A*G*G-3`

Preparation of emulsion

Oil phase

- The reagents (Formulation Aid, Fluid, and Silicone oil) were weighted in a ratio of 4:3:3 based on the composition below (Dow Corning/Sigma Aldrich)
- The oil phase was mixed by vortexing for 10 sec.
- The oil phase was centrifuged at 3000 rpm. Alternatively, the oil phase was allowed to settle for 5 min or until bubbles dissipated, omitting the centrifugation step.

Preparation of the oil phase	
Components	x 1
DC 5225 Formulation Aid (40% w/w) (Dow Corning)	400 µg
DC749 Fluid *Dow Corning (30% w/w)	300 µg
Ar20 Silicon Oil (30% w/w), Sigma Aldrich	300 µg
Sum	1000 µg

Preparation of the MOCK mix			
Stock solutions	Components	End concentrations	x 1
	dH_2O		151.2 µL
10x	Titanium Taq buffer	1x	24 µL
10 mM	$MgSO_4$	2.5 mM	60 µL
10%	BSA	0.1%	2.4 µL
1%	Tween 80	0.01%	2.4 µL
Sum			240 µL

MOCK amplification MIX was stored at room temperature until needed.

Preparation of the aqueous phase			
Stock Solutions	Components	End concentrations	x 1
	dH_2O		85.6 µL
10x	Titanium Taq Buffer	1x	15 µL
50 mM	$MgCl_2$	8 mM	24 µL
10 mM	dNTP`s	1 mM	15 µL
500 µM	Forward-ACH-88bp	9 µM	2.7 µL
5 µM	Reverse-ACH-R-93-SNP_R2	50 nM	1.5 µL
500 µM	Forward-TDII-3	9 µM	2.7 µL
5 µM	Reverse-TDII-OUT	50 nM	1.5 µL

50x	Titanium Taq Polymerase		2 µL	
Sum			150 µL	630 µL

Emulsification in TissueLyser II (BioRad)

- One 5mm steel bead was placed in a 2 ml round-bottom tube.
- 650 µL of the oil phase and 240 µL of MOCK mix were added to the tube. The tube was sealed with Parafilm.
- The tubes were positioned in the TissueLyser II adaptors and the appropriate TissueLyser program was selected mixing the oil and MOCK mix at 25Hz for 5min with a 5mm steel bead. The aqueous phase was added next and a second TissueLyser step was performed at 15Hz for 5mm without steel bead. The second step helped to create larger aqueous emulsion droplets which work as microreactors in the PCR reaction. The surfactant Tween80 and the BSA in the MOCK mix enabled the stabilization of the emulsion.
- 80-100 µL of the emulsion were aliquoted into smaller 200 µL PCR tubes.
- The tubes were placed in the thermocycler and the PCR program outlined below was executed:

<table>
<tr><th colspan="3">Program: BEA72</th></tr>
<tr><td>Temperature</td><td colspan="2">Time</td></tr>
<tr><td>94 °C</td><td colspan="2">2 min</td></tr>
<tr><td>94 °C</td><td>15 sec</td><td rowspan="3">} x 55</td></tr>
<tr><td>65 °C</td><td>15 sec</td></tr>
<tr><td>72 °C</td><td>35 sec</td></tr>
<tr><td>72 °C</td><td colspan="2">2 min</td></tr>
<tr><td>8 °C</td><td colspan="2">forever</td></tr>
</table>

Breakage of emulsion

- Upon completion of the emulsion PCR, the contents of the 200 µL tubes were combined evenly into 2 siliconized 2 ml round-bottom tubes. Approximately 1.5 ml of ethanol were added to each tube.
- The tubes were sealed with parafilm. To break the emulsion 30Hz were applied for 5 min in the TissueLyser II, followed by centrifugation at 17000xg for 2 min.
- The supernatant (SN) was removed and the pellet was rinsed again with ~500 µL ethanol.
- The tubes were filled up with ethanol, homogenized again at 30Hz for 2 min, and centrifuged for 2 min at 17000g.
- The tubes were placed back in the magnetic particle concentrator (MPC), the SN was removed and the tubes were filled up with NXS buffer, and the wash at 30Hz for 1 min in the TissueLyser II was repeated.

- After centrifuging at 17000xg for 1 min, the SN was removed, and the beads were rinsed with TE buffer.
- 500 µL of freshly prepared 0.1M NaOH solution from a 2M stock were added to the bead pellet on the MPC, and the samples were incubated for 1 min at room temperature.
- The NaOH solution was removed and the beads were rinsed again with ~500 µL TE buffer.
- The beads were then either labeled immediately or stored in TE buffer at 4 °C.

Labelling of the beads

- The labelling solution was prepared by combining 41.5 µl Millipore water, 5 µl Titanium Taq buffer, 1 µL dNTPs, 0.5 µl Titanium Taq polymerase, and 0.5 µL of each of the 4 labelling probes.
- The tubes were placed on the magnetic particle concentrator (MPC), the TE buffer was removed and the beads were resuspended in 50 µl labelling solution. Then, the entire content was transferred into a 200 µL PCR tube, and the specified program was run in the thermocycler.
- 500 µl of 1E buffer (10mM Tris-HCl (pH 7.4), 50mM KCl, 2mM EDTA, and 0.01% Triton-X100) were prepared in an empty 1.5 ml reaction tube. After the program has finished, the samples were quickly pipetted into the prepared reaction tubes containing 1E buffer and centrifuged at 9000xg for 1 min.
- The tubes were placed in the MPC and the SN was removed and the pellets were washed with ~500 µl TE buffer.

Array and Scan of the Beads

- To analyze the beads, they were cast within a polyacrylamide matrix (PAA gel) immobilizing them in a monolayer onto a glass slide to maintain positional information over consecutive washing and scanning steps.
- First, the object slides were treated with a layer of Gamma-Methalcryloxypropyl-trimethoxysilan and the polyacrylamide gel was prepared. 4 µl of 20% Rhinohide in Acrylamide (37/1) and 4 µl TE buffer were mixed in 200 µL PCR tubes and TEMED and APS were added shortly before bead arraying.

<table>
<tr><td colspan="3">The beads were resuspended in TE buffer and either arrayed immediately or stored until the arraying step was carried out.
PAA gel preparation</td></tr>
<tr><td>Component</td><td>Percentage</td><td>Amount</td></tr>
<tr><td>TE-Buffer</td><td></td><td>4 µl</td></tr>
<tr><td>Rhinohide in AA (37/1)</td><td>20 %</td><td>4 µl</td></tr>
<tr><td>TEMED</td><td>5 %</td><td>1.25 µl</td></tr>
<tr><td>APS</td><td>0.5 %</td><td>1.25 µl</td></tr>
</table>

- The tubes were placed on the magnetic particle concentrator (MPC) and the TE buffer was removed from the beads. The samples were then removed from the MPC, and air dried for a maximum of 5 min.
- 1.25 µL 5% TEMED and 1.25 µL 0.5% APS were then added to the already prepared Rhinohide-in-Acrylamide/TE buffer-mix.
- The samples were mixed by flicking. Then, 1.9 µL of the mixture were taken out and the PAA gel was added to the beads and mixed by pipetting up and down. The samples were then transferred to the slide.
- The bead drop was covered with a cover slip (12x12mm) and soft pressure was applied to spread the gel over the entire area of the cover slip, creating the desired monolayer. Note: It was important to be cautious to avoid air bubble formation.
- After approximately 5 min the gel was polymerized and the 12x12mm cover slip was removed carefully and a few drops of TE buffer were pipetted on the sample. Then the array was covered with another cover slip (24x40x1mm), the excess liquid was removed with a tissue and the slide was sealed with rubber cement glue.
- The array was scanned with an epifluorescent microscope using ~300 raster positions. Three (or five) images were captured at each raster position in different fluorescent channels as well as the bright field mode with the 12-bit 4K CCD camera. Note: the bright field image was essential to infer the bead area during image analysis.
- If scanning more than two polymorphisms, the array was washed, the fluorophores were stripped off, and the array was labeled with a new set of probes, as explained in the next step (additional probing and imaging of the array).

Additional Probing and Imaging of the Array

- In order to verify the mutants a dye-switch approach was used. The probes on the arrayed beads were stripped off, and the beads were labeled again with another set of probes.
- For this, the dye switch solution was prepared by mixing Titanium Taq buffer 1x, 0.2mM dNTPs, and 1µM each of A592 ACHwt, A488 ACHmut, A532 TDIIwt, and A647 TDIImut, along with Titanium Taq polymerase at 0.02U/µL.
- The glue from the sealed cover slip was removed and subsequently the cover slip (24x40x1mm) was also removed by adding a few drops of TE buffer around the edges (This caused the cover slip to quickly float on the excess TE buffer and enabling a removal without bead loss).
- The array was covered with TE buffer and an in situ PCR block was used to strip off the probes at 94 °C for ~1 min. Note: It was important to ensure that the gel was covered with TE buffer at all times.
- The slide with the array was removed from the in situ block and rinsed with TE buffer.
- The samples were air dried to remove any excess water on the array. The hybridization chamber was placed as centrally as possible on the array and 100 µL dye switch solution was pipetted into the hybridization chamber through the holes at two of the corners. Before sealing the holes with adhesive films, all air bubbles were removed by gently tapping on the surface of the hybridization chamber.
- The slides were placed on the in situ block and incubated for an initial denaturation step at 95 °C for 2 min, followed by a probe annealing at 63 °C for 5 min, and an extension at 72 °C for 5 min, and a final extension at 75 °C indefinitely.

- When removing the slides from the hot in situ block (at ~75 °C), the hybridization chambers were stripped off immediately, and the slides were placed in a Coplin jar filled with 1E buffer to wash off all non-extended allele-specific probes.
- The array was rinsed with TE buffer and covered again with a cover slip (24x40x1mm), and sealed with rubber cement glue.
- When scanning the array for a second or other consecutive time, the slides were placed at the same position on the slide holder of the microscope and Metamorph was used to check how well the first image of the first scan aligns with the bead positions of the second scan. If the offset was too large, the x- and y-positions of the scan area were adjusted for a perfect image overlay.
